# Poor glycemic control impacts heart rate variability in patients with type 2 diabetes mellitus: a cross sectional study

**DOI:** 10.1186/s13104-018-3692-z

**Published:** 2018-08-20

**Authors:** Chris Nadège Nganou-Gnindjio, Camille Maadjhou Mba, Marcel Azabji-Kenfack, Mesmin Y. Dehayem, Liliane Mfeukeu-Kuate, Jean-Claude Mbanya, Eugène Sobngwi

**Affiliations:** 1 Department of Internal Medicine and Specialties, Faculty of Medicine and Biomedical Sciences, University of Yaoundé I, Yaoundé, Cameroon; 2 Cardiology Department, Yaoundé Central Hospital, Yaoundé, Cameroon; 3 Institut Supérieur des Sciences de la Santé, Université des Montagnes, Bangangté, Cameroun; 4 National Obesity Center, Yaoundé Central Hospital, Yaoundé, Cameroon

**Keywords:** Type 2 diabetes patients, Heart rate variability, Glycemic control

## Abstract

**Objective:**

We aimed to determine and compare HRV parameters in poorly and well controlled type 2 diabetes. 54 normotensive type 2 diabetes patients without clinical signs of CAN were enrolled; 29 poorly controlled (HbA1c ≥ 7%) and 25 controls matched for age, sex and BMI. HRV analysis was performed using 24-h ambulatory ECG, with automatic estimation of the time and frequency domain ranges. Comparisons were performed using Mann–Whitney test.

**Results:**

We included 54 participants (26 males) aged 56 years [43–62], with known duration of diabetes 3 years [1–7]. HbA1c was 10.1% [9.1–11.9] vs 5.3% [5.1–6.3] (p < 0.001). Blood pressure was 126 mmHg [121–130] vs 124 mmHg [113–133] in the poorly controlled group and the well-controlled group respectively (p = 0.5). 24-h mean heart rate was significantly higher in poorly controlled vs well controlled patients (79 bpm [77–83] vs 75 bpm [69–79], p = 0.006). In the time domain analysis, markers of the overall variability were lower and thus altered in the poorly controlled group (SDNN: 102 ms [90.5–111.1] vs 112.3 ms [104.4–131.2], p = 0.01 and SDANN 88 ms [72.9–99.7] vs 97.8 ms [91.8–114.5], p = 0.01). The frequency domain analysis showed trends towards lower values of sympathovagal balance markers in the poorly controlled group. Reduced HRV is associated with poorly controlled type 2 diabetes mellitus and may be an early marker in clinical practice.

## Introduction

According to the International Diabetes Federation Diabetes atlas 2017, in Africa over two-thirds (69.2%) of people with diabetes are undiagnosed and 73.5% of all deaths due to diabetes occurred in people under the age of 60; the highest proportion in the world [1]. This may be due to the sustained poor glycemic control which leads to both macro and microvascular complications. Among the microvascular complications, cardiac autonomic neuropathy (CAN) is the most important autonomic neuropathy due to its potential life threatening outcome [2]. Its prevalence is often underestimated and is reported to approximate 17% in type 1 (T1DM) and 22% in type 2 diabetes mellitus (T2DM) [3, 4]. CAN causes primary symptoms which negatively affect the patient’s quality of life, but also secondary symptoms such as silent myocardial infarction [5]. Among patients with diabetes, the 5-year mortality rate is 3 times higher in those with autonomic involvement than in those without [2]. Heart rate variability (HRV) defined as the beat to beat alteration in heart rate may provide a non-invasive tool for the early diagnosis of CAN. It gives quantitative information about the vagal and sympathetic modulation of cardiac functions [6]. Reduction in HRV is the earliest finding of CAN even at subclinical stages. Reduced HRV is a recognized independent cardiovascular risk factor [3]. Little is known whether poor glycemic control in T2DM patients without clinical signs of CAN, could negatively impact cardiac autonomic modulation as indicated by a reduced HRV. The present study aimed to determine the HRV of poorly controlled compared to well-controlled type 2 diabetes mellitus patients in a sub Saharan African population.

## Main text

### Methods

#### Ethical considerations

The study was approved by the Centre Regional Ethical Committee (Authorization No 0110/CRERSHC/2016) and all participants provided written informed consent prior to inclusion.

### Study subjects and design

We conducted a cross sectional study at the National Obesity Centre of the Yaoundé Central hospital in Cameroon. Participants were 29 poorly controlled type 2 diabetes patients (HbA1c ≥ 7%) who were compared at inclusion to 25 well controlled patients (HbA1c ≤ 7%) matched for age, sex and BMI [7]. Patients presenting with clinical signs of CAN such as orthostatic hypotension, resting tachycardia and exercise intolerance, significant renal impairment (eGFR < 60 ml/min), any abnormal ECG finding, treated hypertension or blood pressure ≥ 140/90 mmHg were not included.

### Procedure and investigations

The study was conducted in two visits: an inclusion and exploratory visit. At inclusion visit, eligibility criteria were assessed using a predesigned questionnaire. A detailed clinical examination and venous blood sampling for inclusion biological parameters were performed. The mean of the two blood pressure measurements in a sitting position after 10-min rest using an automatic sphygmomanometer (OMRON^®^ HEM-712 C, OMRON HEALTHCARE, INC. Bannockburn, Illinois 60015 USA) was considered. Blood pressure was also measured following 1 and 3 min of standing in search of orthostatic hypotension. Weight and height were respectively measured to the nearest 0.5 unit and body mass index (BMI) was calculated in kg/m^2^. Patients underwent resting 12-lead electrocardiogram (ECG) recording to exclude findings which may alter heart rate variability analysis such as bundle branch blocks, myocardial infarctions and arrhythmias. Eligible patients were invited the next day to undergo a 24 h ambulatory ECG recording for HRV analysis. They were asked to refrain from heavy physical activity, consumption of caffeinated beverages or any stimulant drink 12 h prior to the visit.

#### Heart rate variability measurement and analysis

The recommendations of the Task Force on HRV were followed for recording long term HRV. We used a 3 channel ECG (EKG recorder Holter monitor TLC 9803). The Holter monitor was connected to the patient’s chest via a series of wired non-invasive pregelified electrodes (Tiga-med Deutschland GmbH, tiga-med EKG-klebeelectroden). These electrodes were firmly fixed to the chest at 5 specific positions. The participants were advised to carry on their usual daily activities except bathing and swimming. They were also asked respect the same cautions as the previous day (refrain from any strenuous exercise, smoke, nor drink caffeinated drinks and alcohol). Electrical signals originating from the heart were recorded via electrodes into a digital flask memory used to store the ECG data. The Holter device was worn by the participants during their normal daily activity for a period of 24 h. Data recorded was uploaded to a computer containing the corresponding software for further processing and analysis. Artifacts and ectopic beats from the RR interval series were cleared up with the help of a cardiologist. HRV was then computed in the time and frequency domain ranges. This automatically provided values for SDNN, SDANN, RMSDD and PNN50 for the time domain range and LF and HF for the frequency domain range.

### Statistical analysis

Data was recorded and analyzed using IBM SPSS statistics for windows version 20.0. Armonk, NY: IBM Corp. Continuous variables were expressed as median with the 25th and 75th percentile while categorical variables in the form of percentages. Non parametrical Mann–Whitney test was used to assess statistical significance. The significance level was set at 5%.

### Results

#### Baseline characteristics

In total 54 consenting type 2 diabetes mellitus patients without clinical signs of CAN were recruited (26 males and 28 females). Table 1 shows that at baseline, median age, socio-demographic characteristics, median duration of diabetes and current treatment were similar in both groups. The median fasting blood glucose and HbA1c was significantly different in the two groups (p < 0.001).

**Table 1:** Comparison of baseline characteristics between the two study groups

Characteristics	Type 2 diabetes mellitus patients		*p* value
	HbA1c ≥ 7%	HbA1c < 7%	
Age (years)	55 [46–61]	54 [46–63]	0.75
Male, n (%)	14 (48.3)	12 (48)	0.6
Known duration of diabetes	3.1 [1.9–5.7]	2.21 [0.95–4.51]	0.09
SBP (mmHg)	126 [121–130]	124 [113–133]	0.53
DBP (mmHg)	74 [67–81]	75 [65–79]	0.73
HR (bpm)	79 [75–84]	78 [70–83]	0.29
Weight (kg)	75 [69–84]	76 [66–83]	0.96
BMI (kg/m^2^)	26.57 [24.56–29.62]	26.47 [24.76–30.09]	0.76
FBG (mmol/l)	10.83 [10.06–11.72]	5.72 [5.33–6.22]	< 0.001
HbA1C (%)	10.10 [9.05–11.91]	5.30 [5.05–6.30]	< 0.001
Serum creatinine (mg/l)	9.00 [7.95–9.80]	9.21 [8.12–10.01]	0.34

Expressed as median [25th–75th percentile] unless otherwise stated

*SBP* systolic blood pressure, *DBP* diastolic blood pressure, *HR* heart rate, *HR* heart rate, *bpm* beats per minutes

#### ECG recordings

Resting ECG findings were not statistically different in both groups.

Table 2 shows that 24-h mean heart rate was statistically higher in the poorly controlled group, while markers of the overall variability of the time domain range such as SDNN and SDANN were statistically lower in the poorly controlled group when compared to the well controlled. There was no significant difference in elements of the frequency domain ranges and sympatho-vagal balance represented by LH/HF.

**Table 2:** Comparison in heart rate variability parameters between the two study groups

Characteristics	Type 2 diabetes mellitus patients		*p* value
	HbA1c ≥ 7% M [Q1–Q3]	HbA1c < 7% M [Q1–Q3]	
24 h mean HR (bpm)	79 [77–83]	75 [69–79]	*0.006*
Markers of sympathetic activity			
SDNN (ms)	102.00 [90.45–111.05]	112.30 [104.40–131.15]	*0.014*
SDANN (ms)	88.00 [72.95–99.70]	97.80 [91.80–114.50]	*0.012*
LF (ms^2^)	259.20 [177.75–632.20]	266.40 [146.60–640.90]	0.822
Markers of parasympathetic activity			
RMSSD (ms)	47.06 [39.70–73.15]	50.60 [27.85–75.90]	0.869
PNN50 (%)	5.70 [3.55–10.25]	6.40 [2.80–11.85]	0.822
HF (ms^2^)	238.90 [112.05–689.85]	276.60 [81.55–698.85]	0.768
Markers of sympatho-vagal balance			
LF/HF	0.95 [0.77–1.42]	0.97 [0.79–1.56]	0.842

Italic values indicate p value < 0.05

*M [Q1–Q3]* median [25th–75th percentile], *HR* heart rate, *bpm* beats per minutes, *ms* millisecond, *ms*^*2*^ millisecond square, *SDNN* standard deviation of all normal to normal interval, *SDANN* standard deviation of average normal to normal interval, *RMSSD* square root of the mean of the sum of the squares of differences between adjacent normal to normal intervals, *PNN50* percentage of normal to normal intervals differing by more than 50 ms, *LF* low frequency, *HF* high frequency

There was no correlation between HRV parameters and the age of participants, duration of diabetes, HbA1c and the weight.

### Discussion

The aim of this study was to compare HRV in a group of poorly controlled type 2 diabetes patients to that of well controlled. Our results show that, HRV parameters of the time domain range (SDNN and SDANN) were significantly lowered thus altered and heart rate higher in poorly controlled patients when compared to their matched controls. On the other hand, there was no significant difference in resting ECG findings such as Sokolov index, QT and calculated QT.

Poorly controlled patients presented with lower HRV measures of the time domain range (SDNN and SDANN) which are components representing the overall variability but mostly the sympathetic activity as previously hypothesized [7, 8]. We did not find significant difference in elements of the frequency domain range such as, LF, HF. This could be explained by the fact that we used the long term recording, and components of the frequency domain methods are adequate when short term recordings are done. The present study however corroborates with Bassi et al. in 2015, which showed that LF, RMSSD and LF/HF of poorly controlled type 2 diabetes mellitus patients were not significantly different from those of the well-controlled type 2 diabetes mellitus patients [9]. Our study showed that 24-h heart rate was significantly higher by 5.3% in the poorly controlled group when compared to the well-controlled group as suggested in shorter term recordings [7]. Since diabetic neuropathy first affects the longest nerve fibers, the earliest manifestation of diabetic CAN tends to be related with vagal nerve damage causing resting tachycardia as the sympathetic tone becomes dominant. Thus, reflecting dysfunction of the autonomic nervous system [3, 10].

Overall, we found in a population with short term duration of known diabetes, that poor glycemic control may affect negatively cardiac autonomic modulation as observed by a reduced HRV in the absence of signs and symptoms of CAN. Reduced HRV is likely to be a very early indicator of cardiac autonomic dysfunction even at subclinical stages. This finding addresses the need to develop a clear policy for early detection and management of CAN and improvement of the availability and affordability of diabetes medications so as to improve glycaemic control in patients with diabetes in this low resource setting.

### Conclusion

The findings of this study suggest that poorly controlled type 2 diabetes mellitus patients are associated with a poor cardiac autonomic modulation as indicated by a reduced heart rate variability.

## Limitations

We used the long-term recording of HRV (24 h ambulatory ECG recording), therefore parameters of the frequency domain range were not appropriate for HRV assessments given that the frequency domain methods are recommended when short term recordings are done. Also, the problem of stationarity which is frequently discussed with long term recordings.

## Abbreviations

HbA1c: glycated hemoglobin
HR: heart rate
HRV: heart rate variability
HF: high frequency
LF: low frequency
ms: milliseconds
RMSSD: root-mean-square of successive differences
SDNN: standard deviation of all normal R–R intervals
SDANN: standard deviation of average normal to normal R–R intervals
T2DM: type 2 diabetes mellitus
